# Haptic curing with endolaser after lens tilt in the Yamane technique

**DOI:** 10.1016/j.ajoc.2024.102049

**Published:** 2024-04-16

**Authors:** James C. Liu, Arsham Sheybani

**Affiliations:** Department of Ophthalmology and Visual Sciences, Washington University School of Medicine, St. Louis, MO, USA

**Keywords:** Yamane technique, IOL tilt, Haptic curing

## Abstract

**Purpose:**

To describe a case of intraocular lens (IOL) tilt following transscleral IOL fixation with the Yamane technique and subsequent correction of tilt using 810-nm endoscopic diode laser.

**Observations:**

Our patient required lens exchange and secondary IOL implantation due to a dislocated 1-piece IOL in the bag causing iris chafe. After routine IOL fixation with the Yamane technique and a Zeiss CT Lucia 602 lens, the IOL tilted significantly in the immediate postoperative period. The lens tilt was subsequently corrected in the operating room using endolaser to melt and reshape (cure) the optic-haptic junction.

**Conclusions:**

IOL tilt is a known complication associated with the Yamane technique, which has seen increasing reports with the Zeiss CT Lucia 602 lens. Using endolaser to reconfigure the tilted optic into the desired orientation is a more novel procedure that can correct the lens tilt without the need for IOL exchange.

## 1 Introduction

Traditional intraocular lens (IOL) implantation necessitates lens capsular and zonular support. When this is absent, flanged intrascleral haptic fixation (FISHF) with the Yamane technique can be used to secure the IOL.^1^ A common lens used in this technique is the Zeiss CT Lucia 602 lens (Zeiss Meditec, Dublin, California, USA) due to its durable polyvinylidene fluoride (PVDF) haptics. However, there have been several recent reports of lens tilt after implantation with this lens.

Lens tilt has been a commonly reported phenomenon and has been ascribed to asymmetric haptic fixation, unequal haptic or scleral tunnel length, and haptic bending or kinking. Often the IOL tilt requires shortening the haptics, re-tunneling of the haptics, or exchange of the tilted lens. A novel technique was described by Drs. Drew Scoles and Jeremy Wolfe where laser treatment is used to reshape and lock the optic-haptic junction in a new configuration.^2^ We describe a similar case of lens repositioning by reshaping the haptics using endolaser. In doing so, the haptic is cured (i.e. melted then set) into the desired orientation.

## 2 Case report

**Fig. 1:**
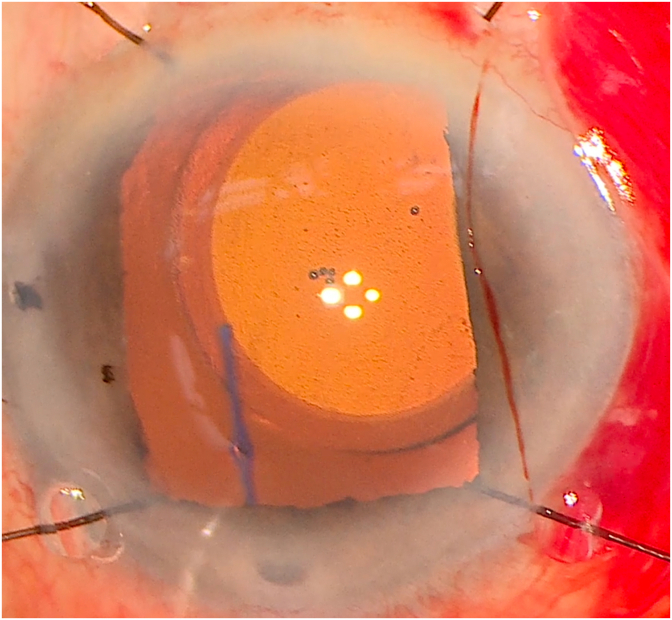
Intraoperative photos of the lens at the end of the initial Yamane procedure, showing superonasal decentration, but planar lens orientation.

**Fig. 2:**
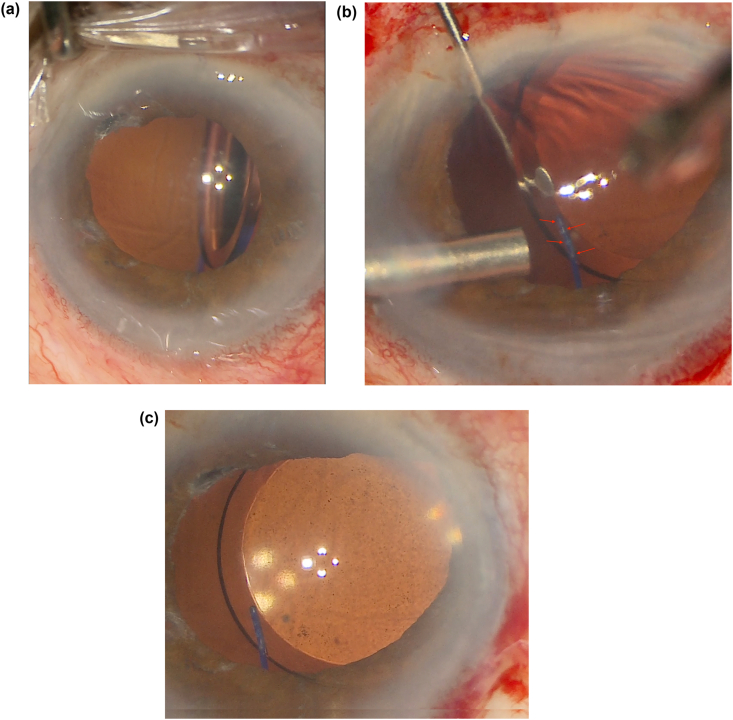
Intraoperative photos of the lens before (a), during (b), and after (c) the repositioning and haptic curing of the scleral-fixated lens. 2a) IOL superonasally decentered and nearly 90 degrees of lens tilt. 2b) Small bubbling (arrows) of the haptic during laser treatment. 2c) IOL superonasally decentered, but with planar lens positioning.

An 83-year-old Caucasian male was referred for inflammation of the right eye. On initial presentation, the patient had count fingers vision with significant iris chafe and anterior uveitis. Clinical exam and ultrasound biomicroscopy (UBM) showed significant lens dislocation. The patient was brought to the operating room for lens repositioning. Due to significant compromise of zonular support, the original lens-bag complex was removed, thorough anterior vitrectomy performed, and a transscleral fixated CT Lucia 602 lens was placed using the Yamane technique. The flanged haptics were noted to be symmetrically oriented 180 apart and equidistant from the limbus. The lens was decentered superonasally, but otherwise in a stable and planar position at the end of the case (Fig. 1). However on postoperative day 1 (POD1), the lens had tilted nearly 90° with the optic oriented perpendicular to iris plane (Fig. 2a). The flanged haptics were securely positioned underneath conjunctiva 180° apart. Vision at that time was again count fingers vision.

On postoperative week 3 (POW3), the patient was taken back into the operating room for lens repositioning (Supplementary Video). The prior main wound and paracentesis were reopened. A limited peritomy was done to expose and visualize both flanged haptics, which again were confirmed to be 180° apart. The haptics were externalized with a 30-gauge needle. The length of each haptic was adjusted, but did not result in any improvement in IOL tilt. At that time, the optic was moved into the anterior chamber to iris capture the lens for temporary stability.

Supplementary video related to this article can be found at https://doi.org/10.1016/j.ajoc.2024.102049

The following is the supplementary data related to this article:

Video 1 Surgical procedure of initial Yamane implantation and subsequent repositioning and haptic curing using 810-nm diode endolaser.1 Video 1

**Fig. 3:**
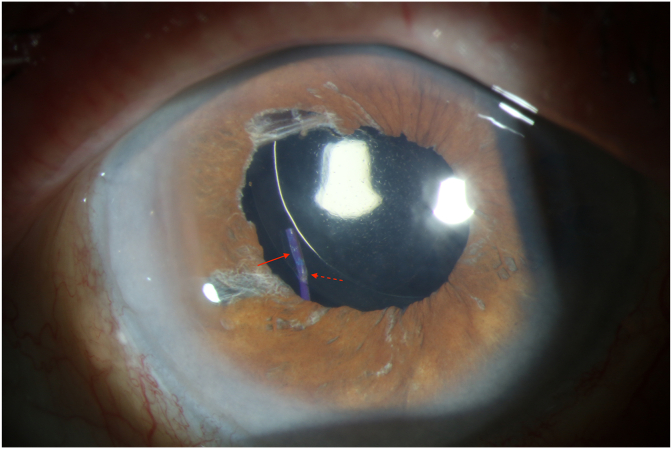
Post-operative week 1 slit lamp photo with superonasal decentration, but planar lens configuration. Slight haptic expansion/bulbing (solid arrow) and haptic bend (dotted arrow) are signs of haptic reconfiguration.

A Kuglan hook was used to provide additional support and the lens was tilted back to an iris plane orientation. 810-nm diode endolaser on continuous treatment at 250mW was then applied to the temporal optic-haptic junction in order to melt and reform (cure) the haptic back into the desired orientation. Small bubbling and whitening of the haptic within its insertion into the optic was noted (Fig. 2b). Treatment duration was titrated based on visual confirmation of small bubbling and whitening of the haptic (approximately 1 second in duration), at which point continuous treatment was stopped. This was done throughout the length of the optic-haptic junction. Similar treatment was applied to the nasal haptic. The lens was positioned back into the sulcus and noted to be parallel to iris plane (Fig. 2c). Subsequently, the lens has remained in a stable, planar configuration on clinical exam (Fig. 3) and UBM with the patient's uncorrected vision improving to 20/100 on POD1 and 20/60 on POW3, which did not improve with pinhole or refraction.

## 3 Discussion

When capsular support is absent, FISHF can be an effective technique to place a secondary lens. With the Yamane technique, the haptics are externalized through the sclera, cautery is used to create a flange at the end of each haptic, then buried into the scleral tunnel.^1^ However, the technique is prone to tilt of the IOL due to its two-point fixation.^3^ Additionally, variability in tunnel length, position of the sclerostomy sites, and differences in eye size can cause strain and stretch of the haptics that may cause the haptics to rotate, bend or kink.

The CT Lucia 602 lens has been proposed as an ideal lens for Yamane fixation due to the flexibility and durability of its polyvinylidene fluoride (PVDF) haptics.^4^ However, recently anecdotal reports of IOL tilt after routine surgery have increased. This is presumed to be secondary to rotation of the haptic at its insertion on the optic. During manufacturing, the haptics of the CT Lucia 602 are normally inserted into the optic manually and held in place with an epoxy mixture.^5^ This naturally introduces some unpredictability in the adhesion strength at the optic-haptic junction, with variability in the manufacturing procedure potentially leading to more cases of lens tilt and instability.

Here we report a newer technique using an endoscopic diode laser probe to treat the optic-haptic junction in order to reverse unwanted lens tilt. We believe the laser serves to melt the material at the optic-haptic junction due to the coagulative/thermal changes from the laser treatment. When the material cools shortly afterwards, the orientation is locked in. In this way, the haptic is cured in place, though microscopic analysis of the structural changes would be needed to confirm this process. Furthermore, long-term stability would need to be assessed with more longitudinal follow-up, which is limited in this case due to patient being lost to follow-up.

This technique offers several advantages in speed, safety, and efficacy. The procedure can utilize the prior cataract wounds, avoid IOL exchange, and could be used prophylactically to improve optic-haptic junction stability and prevent undesired tilt in the postoperative period. While safety of laser application in this setting needs to be considered, we believe that this treatment has relatively low risk to surrounding structures for two reasons: 1) the 810-nm wavelength will be more readily absorbed by the pigmented haptic as opposed to the clear IOL lens complex, and 2) the focal, short duration, and low power settings of thermal laser energy applied reduces any significant absorption from surrounding ocular tissues.^6^^,^^7^

The Yamane technique remains one of the most popular methods for secondary lens implantation. However, increasing reports of lens tilt associated with the technique have prompted the need to develop new methods that can prevent or correct unwanted lens tilt. In our limited experience, haptic curing using 810-nm diode endolaser has been a safe, efficient, and effective procedure for lens repositioning in this setting. While more experience needs to be gained, haptic curing offers a novel way to secure a scleral-fixated lens in place, correcting unwanted lens tilt associated with the Yamane technique.

## Patient consent

Written consent to publish this case has not been obtained. This report does not contain any personal identifying information.

## Funding

There were no funds allocated to the realization of this clinical case.

## Authorship

All authors attest that they meet the current ICMJE criteria for Authorship.

## CRediT authorship contribution statement

**James C. Liu:** Writing – review & editing, Writing – original draft, Methodology, Conceptualization. **Arsham Sheybani:** Supervision, Conceptualization.

## Declaration of competing interest

The authors declare that they have no known competing financial interests or personal relationships that could have appeared to influence the work reported in this paper.
